# Eczema Herpeticum Misdiagnosed as Facial Cellulitis in an African American Male

**DOI:** 10.7759/cureus.58328

**Published:** 2024-04-15

**Authors:** Pallavi Lanka, Jason R Woloski

**Affiliations:** 1 Family Medicine, Geisinger Health System, Wilkes-Barre, USA; 2 Family Medicine Residency Program, Geisinger Health System, Geisinger Commonwealth School of Medicine, Wilkes-Barre, USA

**Keywords:** facial cellulitis, hsv keratitis, eczema herpeticum, atopic eczema, herpes simplex-1 (hsv-1)

## Abstract

Eczema herpeticum (EH) is a cutaneous manifestation of disseminated herpes simplex virus, commonly observed in patients with active eczema. The condition often presents with systemic symptoms, including fever and fatigue, alongside vesiculopustular skin lesions. This case report describes a 19-year-old male with active eczema who was misdiagnosed with facial cellulitis in the emergency and inpatient setting in a community tertiary hospital. With worsening rash and development of ocular symptoms, the diagnosis was reconsidered to be more consistent with EH with herpetic keratitis, which improved with antiviral treatment. This report shows the significance of maintaining a high index of suspicion for EH in patients with eczema and the potential consequences of misdiagnosis and delay in treatment. It aims to enhance clinician awareness of EH and promote a broader differential for unusual presentations of common dermatological and ophthalmologic conditions, especially when caring for patients with limited access to specialist evaluation.

## Introduction

Eczema herpeticum (EH), or Kaposi’s varicelliform eruption, is a cutaneous manifestation of disseminated herpes simplex virus (HSV) infection. EH is often seen in patients with eczema and can be life-threatening [1-3]. EH is characterized by vesiculopustular lesions over areas of active or quiescent eczema, often accompanied by systemic symptoms such as fever and fatigue [1,4,5]. This case report presents a unique instance of EH in a patient who presented to the hospital with fever, syncopal episode, facial rash, and lymphadenopathy after failing treatment as an outpatient for a misdiagnosed suspected eczema flare with presumed facial cellulitis. A key focus of this report is the careful navigation of corticosteroid use in the context of EH. This is a critical topic due to the widespread use of corticosteroids in the management of eczema, but potentially detrimental effects in the setting of concurrent HSV infections [6].

Furthermore, this case presentation discusses the importance of early recognition and appropriate management of EH, as well as the necessity of considering EH in the differential diagnosis of patients with eczema and systemic symptoms [3,7]. The case shows the significance of maintaining a high index of suspicion for EH in patients with eczema, particularly when presenting with unusual or severe symptoms, and the potential consequences of delayed diagnosis. Lastly, this case report aims to enhance awareness and understanding of EH among primary care clinicians. In turn, physicians can provide more effective patient care through the careful consideration of ophthalmic corticosteroid use and a broader approach to unusual presentations of common dermatological conditions.

## Case presentation

**Figure 1:**
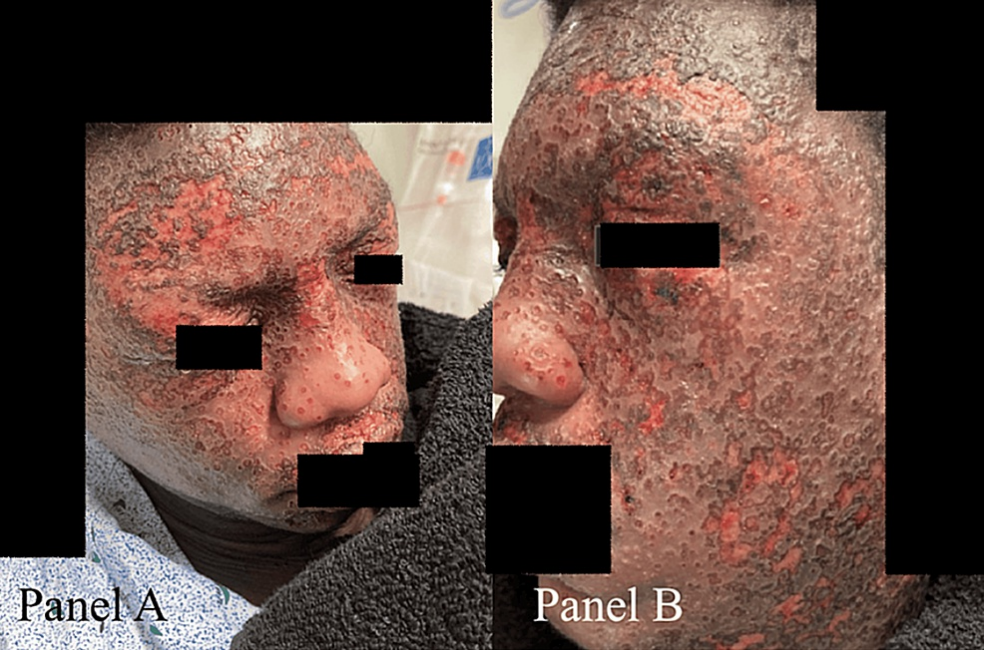
Facial rash on day one of admission displaying widespread erythematous eroded crusted vesicles over the face. A: Front view. B: Side view.

**Figure 2:**
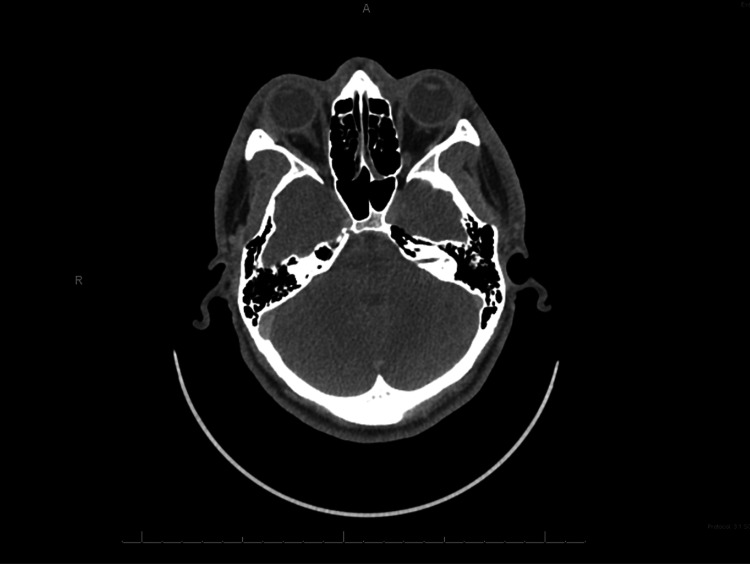
CT of the head and neck showing dermal thickening and subjacent subcutaneous stranding through the anterior face consistent with cellulitis.

A 19-year-old male with a past medical history significant for uncomplicated mild intermittent asthma and unknown history of eczema presented initially to the urgent care department with a fever of 103.7°F (39.8°C) and worsening facial rash with erythematous eroded crusted vesicles diagnosed in the urgent care as an eczema flare with underlying cellulitis. He was initially discharged home with a seven-day course of sulfamethoxazole-trimethoprim 800-160 mg twice daily and bacitracin ointment to be applied to the face three times per day.  After two days, the patient returned to the emergency department with worsening facial rash, facial and neck pain, lymphadenopathy, and pain with movement of the jaw (Figure 1). A CT of the face and neck was obtained which showed diffuse dermal thickening and subjacent subcutaneous stranding through the anterior face and neck with submandibular and submental adenopathy, consistent with diffuse facial and anterior neck cellulitis. There were no signs of abscess, orbital cellulitis, or deeper space infection (Figure 2). Labs on admission showed normal white count and stable electrolyte levels but with slightly elevated creatinine. The procalcitonin level was 0.11 ng/mL, and C-reactive protein and erythrocyte sedimentation rate were 78 mg/L and 74 mm/hour, respectively, which were reassuring for bacteremia and sepsis, though definitive blood and wound cultures were obtained, given low sensitivity and predictive value of procalcitonin [8,9]. The patient was admitted with a diagnosis of worsening eczema with superimposed facial cellulitis and was started on broad-spectrum antibiotics with intravenous (IV) vancomycin and piperacillin/tazobactam, given radiographic evidence of cellulitis, normal saline at 100 mL/hour, and symptom control which included acetaminophen and ketorolac as needed for pain. Consults were placed for Dermatology, Infectious Disease, and Wound care as well.

**Figure 3:**
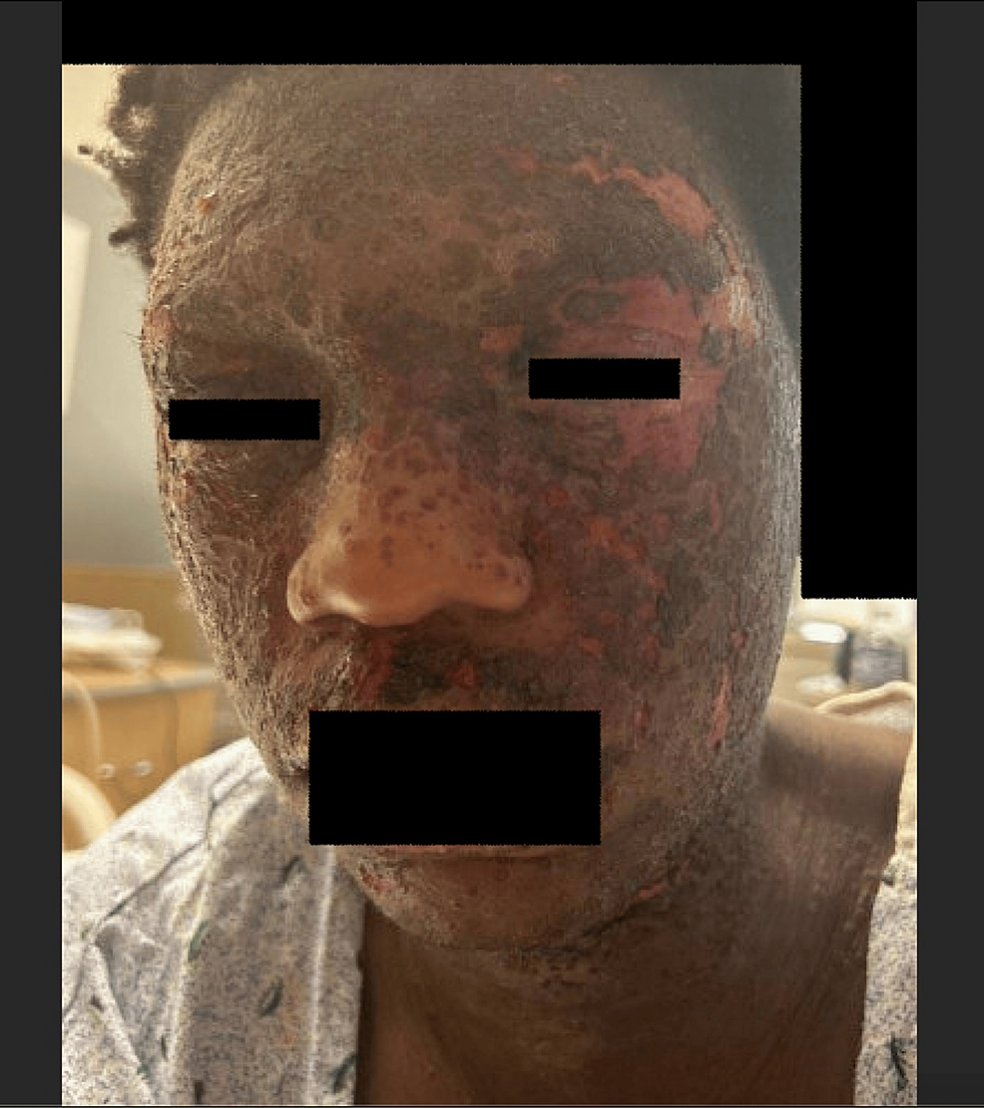
Facial rash on day two of admission with increased periorbital swelling and neck involvement.

Within 24 hours of admission, his facial rash worsened, and he reported new photophobia and blurred vision, despite IV antibiotic treatment. The following day labs showed a stable white count with stable renal function and electrolytes. He exhibited no evidence of airway compromise and was tolerating diet without issues. The physical examination additionally showed worsening periorbital swelling and new conjunctival injection of both eyes (Figure 3). Dermatology was consulted via chart review, who determined that the patient had findings consistent with EH and recommended obtaining viral swab cultures and starting IV acyclovir. Viral swabs were positive for HSV, supporting the presumptive diagnosis. Infectious Disease recommended IV vancomycin as the patient tested positive for a methicillin-resistant *Staphylococcus aureus* in the nares from an admission screen. The patient was started on tobramycin-dexamethasone eye drops at this time given the new conjunctival injection. Later that day, Ophthalmology was consulted to evaluate the patient for eye involvement. The ophthalmic examination showed possible early dendritic formations on fluorescein staining of the left eye. No viral swabs of conjunctiva were obtained due to the diagnosis of EH and characteristic corneal lesions. Recommendations were made to immediately stop the steroid-containing eye drops and to start antiherpetic eye drops due to active HSV corneal lesions. The patient was started on trifluridine eye drops every two hours to both eyes for a diagnosis of herpetic keratitis overlying EH. Wound care recommended leaving facial lesions open to air to allow for drainage.

**Figure 4:**
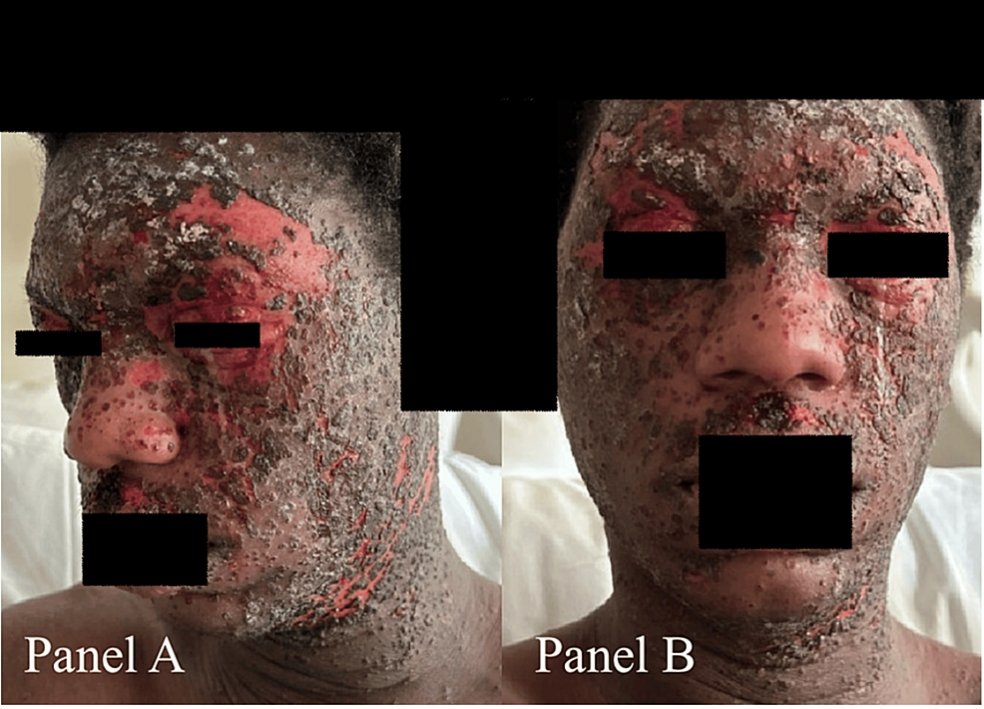
Facial rash on day three of admission with decreased swelling. A: Side view. B: Front view.

The patient showed significant improvement following the adjustments to his initial treatment over the following three days in the hospital. His rash showed improvement with the resolution of his pain (Figure 4). Additionally, his visual acuity improved back to his baseline vision and his periorbital swelling completely resolved. Vitals were monitored every four hours, the last reported fever was at the time of admission with a maximum temperature of 102.6°F, the following day the patient was afebrile and remained so until discharge. His discharge medication regimen included doxycycline 100 mg twice per day for 10 days and valacyclovir 1,000 mg twice per day for seven days. The patient was instructed to continue trifluridine drops to both eyes every two hours until evaluated by Ophthalmology as an outpatient for follow-up.

**Figure 5:**
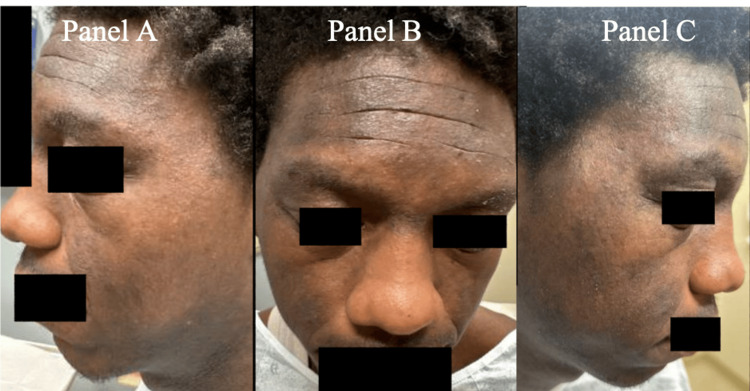
One-year follow-up showing full recovery from eczema herpeticum. A: Left-side view. B: Front view. C: Right-side view.

The patient received an outpatient follow-up with Dermatology one year following hospitalization, which showed significant improvement in his dermatitis (Figure 5). With the resolution of his EH, but recurrent flares of his atopic disease in multiple areas including dorsal hands, elbows, knees, and neck, it was recommended that the patient be started on dupilumab. The patient has not had any formal outpatient follow-up with Ophthalmology or Optometry but has not reported any blurred vision or ocular pain otherwise, which would suggest resolution of his keratitis.

## Discussion

This case highlights the importance of proper diagnosis in patients with severe acute dermatologic conditions. Despite later data suggesting that cellulitis was not present, the patient was initially treated for bacterial cellulitis with underlying eczema flare based on clinical findings of erythematous edema with crusted skin lesions and radiographic findings of dermal thickening and subcutaneous stranding. With the sudden progression of symptoms and the lack of improvement with broad-spectrum antibiotics, further evaluation was initiated, including dermatology and ophthalmology consults. The patient was determined to have untreated disseminated HSV infection in addition to his superimposed cellulitis. The case was further complicated with ocular involvement that was initially treated with steroid eye drops to help with orbital swelling which was initially attributed to facial cellulitis and worsening of EH rash. The steroid eye drops were promptly discontinued following ophthalmologic evaluation identifying early dendritic lesions on fluorescein exam.

The first learning objective from this case is the importance of keeping a broad differential diagnosis with patients presenting with atypical and severely progressive rashes, especially those with a history of eczema and atopy. Dermatologic conditions such as EH might be obvious for dermatologists; however, patients with acute presentations might present to outpatient urgent care and primary care clinics where knowledge regarding such conditions is limited. Prompt identification of EH and appropriate antiviral treatment is important to maximize patient recovery and reduce complications [2]. As seen in this case, the spread of this infection and the severity of symptoms were rapidly progressive, likely due to incorrect diagnoses and delay in antiviral therapy initiation. Early dermatologic evaluation for clinical manifestations and viral swabs of lesions is essential to identify and treat EH to prevent progression and undesired outcomes. Bacterial and viral cultures of seeping lesions with any discharge can help confirm the diagnosis [3].

Although these conditions can be treated with steroids, the provider must be cautious not to prescribe steroids in the setting of potential viral rash with ocular involvement due to the increased risk of chronic scarring of the cornea. While the patient did not have ocular involvement on initial presentation, prevention and treatment of ocular involvement of EH is also a factor that clinicians should consider. HSV infections can involve the external eye, including the conjunctiva and the cornea [10,11]. HSV keratitis is an especially concerning complication that can leave patients with chronic scarring of the corneal stroma, corneal melting, perforation, and permanent visual damage if left untreated [6,10,12]. While topical steroids are an effective treatment for many ocular conditions to reduce inflammation, providers should maintain caution with infectious processes. The question of steroid eye drop use in herpetic infections is nuanced and patients may ultimately benefit from ophthalmologic consultation before starting topical steroid drops. Steroid eye drops are contraindicated in herpetic keratitis with active lesions, specifically dendritic staining under fluorescein dye of the cornea [10,12]. Treatment with steroid drops in these cases may significantly worsen the infection and lead to severe damage to the cornea with overall worse visual outcomes [10,12]. The treatment of choice for these patients is topical antiherpetic eye drops with systemic antiviral treatment in this case for EH. Steroid eye drops are used following the resolution of active herpetic corneal lesions to reduce inflammation and scarring.

Lastly, if immediate access to specialty care for dermatology and ophthalmology is not available, the primary team should have a lower threshold to consider transferring the patient from tertiary centers to seek more timely and in-person care. EH can quickly progress and pose significant morbidity to patients if diagnosis and proper treatment are delayed. While the dermatology consult was placed promptly, relying on images and chart review delayed and limited care. If the patient had been seen by dermatology in person on the day of the initial presentation, his rapid progression and ocular involvement might have been prevented.

## Conclusions

This case report emphasizes the importance of maintaining a broad differential diagnosis in patients presenting with atypical and progressive rashes, particularly in those with a history of eczema and atopy. Early dermatologic evaluation, bacterial and viral cultures of lesions, and timely initiation of antiviral therapy can prevent the progression of the infection and potential complications, such as ocular involvement. If ocular involvement is suspected, prompt evaluation by ophthalmology is essential.

This case also serves as a reminder for clinicians to exercise caution when using ophthalmic corticosteroids in patients with EH, as they may exacerbate HSV infections. In cases of suspicion of ocular involvement, ophthalmologic consultation is essential to guide the use of steroid eye drops and to initiate appropriate treatment for herpetic keratitis. By increasing awareness of EH and the complexities surrounding its management, clinicians can contribute to better patient care and minimize the patient’s risk for adverse outcomes.
